# The Framingham risk score is associated with incident frailty, or is it?

**DOI:** 10.1186/s12877-021-02387-4

**Published:** 2021-07-31

**Authors:** Hui Shi, Mei-Ling Ge, Birong Dong, Qian-Li Xue

**Affiliations:** 1 The Center of Gerontology and Geriatrics (National Clinical Research Center for Geriatrics), West China Hospital, Sichuan University, Chengdu, China; 2 Department of Medicine, Division of Geriatric Medicine and Gerontology, School of Medicine, Johns Hopkins University, 2024 E. Monument Street, Suite 2-700, Baltimore, MD 21205 USA; 3 Center on Aging and Health, Johns Hopkins Medical Institutions, Baltimore, Maryland USA

**Keywords:** Framingham risk score, Cardiovascular disease, Frailty, Cohort study

## Abstract

**Backgrounds:**

Cardiovascular disease (CVD) risk factors are individually associated with frailty. This study examined whether Framingham CVD risk score (FRS) as an aggregate measure of CVD risk is associated with incident frailty among Chinese older adults.

**Methods:**

This study used data from the China Health and Retirement Longitudinal Study. A sample of 3,618 participants aged 60 to 95 years and without CVD at baseline were followed for four years. FRS was calculated at baseline. Frailty status was defined as not-frail (0–2 criteria) or frail (3–5 criteria) based on the physical frailty phenotype consisting of five binary criteria (weakness, slowness, exhaustion, low activity level, and weight loss). After excluding subjects who were frail (*n* = 248) at baseline, discrete-time Cox regression was used to evaluate the relationship between FRS and incident frailty.

**Results:**

During a median follow-up of 4.0 years, 323 (8 %) participants developed CVD and 318 (11 %) subjects had frailty onset. Higher FRS was associated with greater risk of incident frailty (HR: 1.03, 95 % CI: 1.00 to 1.06) after adjusting for education, marital status, obesity, comorbidity burden, and cognitive function. This association however was no longer significant (HR: 1.00, 95 % CI: 0.97 to 1.03) after additionally adjusting for age. These findings remained essentially unchanged after excluding subjects with depression (*n* = 590) at baseline or incident CVD (*n* = 323) during the 4-year follow-up.

**Conclusions:**

The FRS was not independently associated with incident frailty after adjusting for chronological age. More research is needed to assess the clinical utility of the FRS in predicting adverse health outcomes other than CVD in older adults.

**Supplementary Information:**

The online version contains supplementary material available at 10.1186/s12877-021-02387-4.

## Background

By 2050, it is expected that one in four Chinese citizens will be 65 years of age or older [1]. Population aging is believed to be responsible for the growing prevalence of cardiovascular disease (CVD) and frailty in China. Frailty and CVD are two common and often coexisting conditions in the elderly that share many risk factors (hypertension, smoking, obesity, diabetes, and dyslipidemia), and exert a substantial influence on clinical outcomes [2, 3] (e.g., disability, sarcopenia and dementia). The generalized Framingham risk score (FRS) [generalized FRS; 2008] [4] is a standard tool for assessing the 10-year risk of CVD events (i.e., coronary heart disease, cerebrovascular disease, peripheral vascular disease, heart failure). In 2015, an updated FRS [2015] calculator (https://www.thecalculator.co/health/Framingham-Risk-Score-Calculator-for-Coronary-Heart-Disease-745.html) was published. It has been shown that higher FRS is associated with multiple adverse health outcomes, e.g., incident chronic kidney disease (CKD) [5], sarcopenia [6], and cognitive decline [7]. Frailty is a geriatric syndrome characterized by reduced physiological reserve and increased vulnerability for poor recovery of homeostasis after a stressor event [8]. Frailty poses a high risk of developing negative health outcomes including incident disability [9], falls [10], fracture [11], and mortality [12].

Several studies have shown cross-sectional associations between frailty and CVD risk factors [13–16]. It is therefore expected that the FRS may be useful in identifying individuals at increased risk of developing frailty. In addition, researches in the Whitehall II prospective cohort study and the English Longitudinal Study reported that higher sex-specific FRS was associated with increased risk of incident frailty [17, 18] as defined by the physical frailty phenotype [19]. Given that age is a part of the FRS algorithm and frailty is age-related, the degree to which the association between FRS and frailty is driven by age in older adults is unknown. To address this question, we examined the predictive value of FRS versus age for incident frailty among community-dwelling Chinese older adults enrolled in the China Health and retirement Longitudinal Study (CHARLS).

## Methods

### Study population

The study sample consists of Chinese residents aged ≥ 45 years who were recruited from 28 provinces in China. A total of 17,708 residents were interviewed at wave 1 (baseline) between 2011 and 2012, and were followed every 2 years thereafter. All participants gave informed consent; ethical approval for all the CHARLS waves was granted from the Institutional Review Board (IRB) at Peking University. The IRB approval number for the main household survey, including anthropometrics, is IRB00001052-11015; the IRB approval number for biomarker collection, was IRB00001052-11014. Further details about the recruitment strategy, design, and sampling approaches of the CHARLS have been supplied elsewhere [20].

**Fig. 1:**
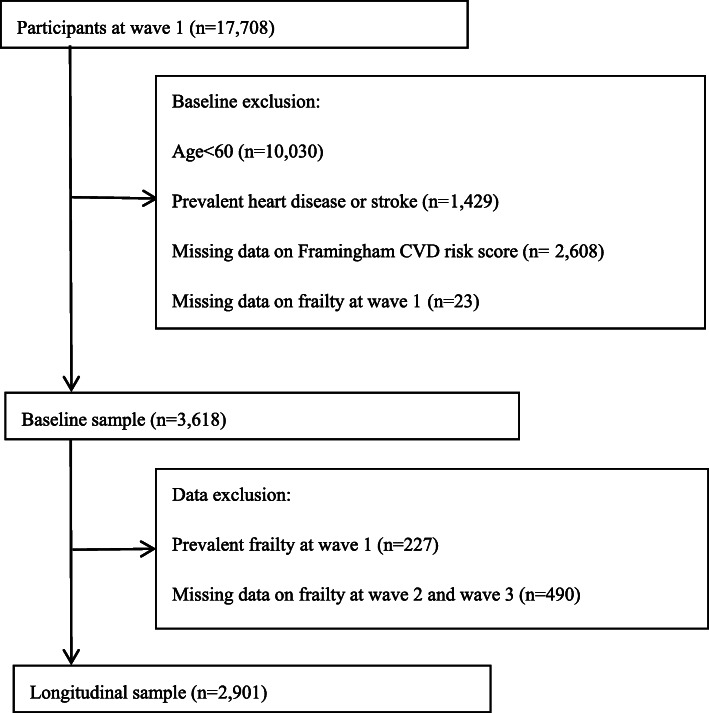
Flow chart of Participants through the study

We utilized the Framingham Risk factors measured at baseline to predict the risk of developing frailty at wave 2 (2013–2014) and wave 3 (2015–2016). We included participants who (i) were 60 years of age or old, (ii) had complete data on FRS and frailty, and (iii) no history of CVD at baseline (see flowchart of sample selection in Fig. 1).

### FRS at baseline

Data on the FRS components (age, HDL, total cholesterol, systolic blood pressure, treatment for hypertension, smoking and diabetes) was taken from wave 1. Blood samples were collected from vein in fasting state (fasting more than 12 h) by professional staff from the Chinese Center for Disease Control and Prevention (China CDC). Fasting glucose and lipid profiles (e.g., total cholesterol, high-density lipids cholesterol, low-density lipids cholesterol) were measured by the enzymatic colorimetric test [20, 21]. Systolic blood pressure (SBP) was measured three times using an Omron HEM-7200 blood pressure monitor with the participant seated [20]. Antihypertensive therapy was defined by self-report of currently taking Chinese traditional or western medication to treat or control hypertension. Current smoking (yes/no) was defined by self-report. Prevalent diabetes mellitus was defined based on self-report of doctor-diagnosed diabetes, use of diabetes medication or insulin injections, a hemoglobin A1C level ≥ 6.5 %, or a baseline fasting plasma glucose level > 126 mg/dl. Total FRS is obtained by summing up the points from all risk components. A higher FRS indicates a greater risk for future CVD events [4].

### Frailty

Frailty was measured using a modified version of the physical frailty phenotype (PFP) [19, 22]. This measure comprised of five binary criteria: weakness, slowness, exhaustion, low activity level and weight loss. Those with 3 or more criteria were deemed frail.

#### Weakness

Grip strength by sex and quartiles of body mass index (BMI; kg/${\text{m}}^{2}$): Weakness was defined, using maximum handgrip strength of either hand (two trials for each; measured in a standing position), as ≤ 20th percentile of the weighted sample distribution, adjusting for sex and body mass index (BMI). Subjects who were tried but unable to perform, could not participate due to health reasons or felt unsafe were considered meeting the weakness criterion.

#### Slowness

Gait speed over a 2.5-meter course by sex and median standing height (cm): Slowness was defined, using the average of two-timed walk tests over a 2.5-meter course, as being ≤ 20th percentile of the weighted sample distribution, adjusting for sex and height via a residual-based approach described previously. Subjects who were unable to walk or felt unsafe were considered meeting the slowness criterion.

#### Exhaustion

Exhaustion is characterized by two questions from the modified 10-item Center for Epidemiological Studies-Depression scale. Two questions were, “I could not get going” and “I felt everything I did was an effort”. If Participants answered “a moderate amount of time (3–4 days)” or “most of the time (5–7 days)” to either of these items, they met the criteria for exhaustion.

#### Low activity level

Physical activity was measured using the International Physical Activity Questionnaire Short Form (IPAQ-SF) [23]. Participants who self-reported that they did not walk ≥ 10 min continuously during a usual week were considered sedentary.

#### Weight loss

Weight loss was defined as self-reported weight loss of 5 or more kilograms in the previous year (at wave 1) or loss of ≥ 10 % since last wave (at wave 2 and wave 3) or BMI ≤ 18.5 kg/m^2^.

### Ascertainment of incident CVD events

Incident CVDs were assessed over the follow-up period (wave 2 to wave 3), using the following standardized questions: “Have you been told by a doctor that you have been diagnosed with a heart attack, coronary heart disease, angina, congestive heart failure, or other heart problems?” or “Have you been told by a doctor that you have been diagnosed with a stroke?” Participants who reported heart disease or stroke during the follow-up period were defined as having incident CVD [24, 25].

### Covariates at baseline

Demographics included age, sex, education, marital status, and current residence location. Body mass index (BMI) was calculated as weight (kilograms) divided by height (meters) squared, then classified into non-obese (BMI < 28 kg/m^2^) and obese (BMI ≥ 28.0 kg/m^2^) [26]. Cognitive function was evaluated by the Telephone Interview of Cognitive Status (TICS) [27], with a score ranging 0–21 and higher score representing better cognitive function. Depression symptoms were assessed using the modified 10-item CES-D scale [20] after excluding two items used to define exhaustion (a frailty criterion), with a total score of 12 or higher indicating depression [28]. Comorbid disease burden was measured by the total number of chronic conditions including cancer (excluding skin cancers), chronic lung diseases, liver disease, kidney disease, stomach or other digestive disease, arthritis/rheumatism, and asthma.

### Statistical analysis

We used ANOVA and chi-square tests to compare the distributions of continuous and categorical variables respectively by baseline frailty status (not-frail vs. frail). After excluding prevalent frailty at baseline, we conducted the discrete-time Cox regression (DTSA) to examine the association between the components of FRS measured at baseline and frailty incidence during the follow-up. When examining the association between FRS and incident frailty, we fit three nested models with increasing number of confounders: sex only (Model 1); sex, education, marital status, obesity, comorbidity burden, and cognitive function (Model 2); and those of Model 2 plus age (Model 3).

We conducted three sensitivity analyses. First, in order to examine whether the association between FRS and incident frailty was driven by incident CVD, we repeated the analysis after excluding incident CVD cases over the follow-up period. In addition, to eliminate the influence of depressive symptoms on frailty measurement, we refit the model by restricting the analysis to those without depression [28]. Third, to explore the effect of competing mortality, we modeled mortality and/or incident frailty as a composite outcome. Finally, in order to determine whether different versions of the FRS would impact the association between FRS and incident frailty, baseline FRS were calculated using both the generalized FRS [2008] and the updated FRS [2015] algorithm.

*P*-value < 0.05 was considered statistically significant, and all analyses were performed using Stata 15.1 (Stata Corp, College Station, TX).

## Results

### Sample description

Table 1 compares the baseline characteristics of 3,618 participants by baseline frailty status. There were 3,313 (91.6 %) classified as not-frail and 305 (8.4 %) as frail. Compared with the not-frail, frail participants were more likely to be older, have worse cognitive function, more depressive symptoms, and greater comorbidity burden. The FRS components except HDL cholesterol did not significantly vary by frailty status at baseline.

**Table 1:** Characteristics of study sample by baseline frailty status (*n*= 3,618^a^)

Characteristic	All	Frailty status at baseline		
		Not-frail	Frail	*P* Value
		*n*=3,313(91.6)	*n*=305 (8.4)	
Age (years), mean (SD)	67.7 (6.5)	67.4 (6.2)	71.9 (7.7)	<0.001
Sex, n(%)				
Male	1,835 (50.7)	1,686 (50.9)	149 (48.9)	0.496
Female	1,783 (49.3)	1,627 (49.1)	156 (51.2)	
Framingham risk score, mean (SD)	15.1 (4.5)	15.1 (4.5)	15.55 (4.7)	0.096
Total cholesterol (mg/dl), mean (SD)	194.6 (38.5)	194.8 (38.3)	192.1 (40.3)	0.245
HDL cholesterol (mg/dl), mean (SD)	52.3 (15.7)	52.16 (15.6)	54.2 (16.2)	0.031
SBP (mm Hg), mean (SD)	135.1 (22.8)	135.0 (22.5)	136.6 (25.8)	0.236
Antihypertensive treatment, n (%)				
No	2,870 (79.3)	2,620 (79.1)	250 (82.0)	0.234
Yes	748 (20.7)	693 (20.9)	55 (18.0)	
Smoking, n (%)				
No	2,467 (68.2)	2,252 (68.0)	215 (70.5)	0.366
Yes	1,151 (31.8)	1,061 (32.0)	90 (29.5)	
Diabetes, n (%)				
No	3,061 (84.6)	2,796 (84.4)	265 (86.9)	0.249
Yes	557 (15.4)	517 (15.6)	40 (13.1)	
Education, n (%)				
Illiterate	1,399 (38.7)	1,234 (37.3)	165 (54.1)	<0.001
Elementary school	1,643 (45.4)	1,524 (46.0)	119 (39.0)	
Middle school	410 (11.3)	391 (11.8)	19 (6.2)	
High school or above	164 (4.5)	162 (4.9)	2 (0.7)	
Current residence, n (%)				
Rural	2,464 (68.1)	2,230 (67.3)	234 (76.7)	0.001
Urban	1,154 (31.9)	1,083 (32.7)	71 (23.3)	
Marital status, n (%)				
Married	2,899 (80.1)	2,689 (81.2)	210 (68.9)	<0.001
Divorced	47 (1.3)	42 (1.3)	5 (1.6)	
Widowed	638 (17.6)	552 (16.7)	86 (18.2)	
Never married	34 (0.9)	30 (0.9)	4 (1.3)	
Obese, n (%)				
No	3,301 (91.2)	3,022 (92.6)	279 (93.9)	0.400
Yes	259 (7.3)	241 (7.4)	18 (6.1)	
Cognitive function^b^, mean (SD)	10.15 (4.2)	10.35 (4.1)	7.79 (4.0)	<0.001
Depressive symptoms scores ^c^, mean (SD)	7.68 (5.3)	7.37 (5.2)	11.04 (5.4)	<0.001
Comorbidity burden ^d^, mean (SD)	1.35 (1.3)	1.31 (1.3)	1.80 (1.4)	<0.001

Abbreviations: *SBP* systolic blood pressure, *CVD* cardiovascular disease, *SD* standard deviation, *HDL* high density lipoprotein cholesterol

^a^before excluding missing data on frailty at follow-up waves (*n*=490) and prevalent frailty at baseline (*n*=227)

^b^Cognitive function was measured by the modified mini-mental status examination

^c^Depressive symptoms was measured by the 10-item Center for Epidemiologic Studies Depression Scale

^d^Comorbidity includes cancer[excluding minor skin cancers], chronic lung diseases, liver disease, kidney disease, stomach or other digestive disease, arthritis/rheumatism and asthma

### Association between FRS and incident frailty

The association between each component of the generalized FRS [2008] at baseline and incident frailty during the follow-up is presented in Table 2. Older age, lower level of total cholesterol, and smoking were associated with greater risk of incident frailty in both sex-adjusted and fully-adjusted models. On the other hand, a one SD increase in total cholesterol predicted a 24.0 % (Hazard Ratio [HR] 0.76, 95 % CI 0.66–0.89) and 17.0 % (HR 0.83, 95 % CI 0.71–0.96) lower risk of incident frailty in sex-adjusted and fully-adjusted models, respectively. These associations were not statistically different by sex except for antihypertensive treatment (results not shown), where the association was weaker among females compared to males (*P* = 0.033).

**Table 2:** Association between each cardiovascular disease risk factor at baseline (wave 1) and incident frailty during the follow-up (wave2-wave3). (*n*=2,901)

	Sex-adjusted Model ^b^		Fully-adjusted Model ^c^	
	HR (95% CI)	*P* value	HR (95% CI)	*P* value
Age (years)	1.09 (1.07 to 1.11)	<0.001	1.08 (1.06 to 1.10)	<0.001
Sex				
Male	1 (ref)		1 (ref)	
Female	1.73 (1.31 to 2.27)	<0.001	1.41 (1.00 to 1.98)	0.049
Total cholesterol (mg/dl) ^a^	0.76 (0.66 to 0.89)	<0.001	0.83 (0.71 to 0.96)	0.006
HDL cholesterol (mg/dl) ^a^	1.08 (0.96 to 1.22)	0.155	1.05 (0.93 to 1.19)	0.544
Systolic blood pressure (mm Hg) ^a^	0.98 (0.85 to 1.12)	0.610	0.93 (0.82 to 1.07)	0.299
Antihypertensive treatment				
No	1 (ref)		1 (ref)	
Yes	1.17 (0.89 to 1.55)	0.266	1.31 (1.96 to 1.79)	0.087
Smoking				
No	1 (ref)		1 (ref)	
Yes	1.37 (1.03 to 1.83)	0.031	1.42 (1.04 to 1.95)	0.029
Diabetes				
No	1 (ref)		1 (ref)	
Yes	1.06 (0.78 to 1.43)	0.731	1.17 (0.84 to 1.62)	0.358

*HR* hazard ratio, *CI* confidence interval

^a^HR per SD (Total cholesterol:38.46, HDL cholesterol: 15.69, Systolic blood pressure: 22.80) increase

^b^Adjusted for sex

^c^Adjusted for sex, education, marital status, obesity, comorbidity burden, and cognitive function score

The association between the FRS at baseline and incident frailty is shown in Table 3. There was a significant association between increasing FRS at baseline and increasing risk of incident frailty after adjusting for sex (HR 1.03, 95 % CI 1.01–1.06; Model 1). Further adjustment of education, marital status, obesity, comorbidity burden, cognitive function had little impact (HR 1.03, 95 % CI 1.00-1.06). The association however became insignificant after additionally adjusting for age (HR 1.00, 95 % CI 0.97–1.03; Model 3). The results showed similar trends when the updated FRS [2015] was used instead of the generalized FRS [2008].

**Table 3:** Associate between the Framingham Risk Score (FRS) at baseline (wave 1) and incident frailty during the follow-up (wave2-wave3)

	Model 1 ^a^		Model 2 ^b^		Model 3 ^c^	
	HR (95% CI)	*P* Value	HR (95% CI)	*P* Value	HR (95% CI)	*P* Value
Generalized FRS [2008](*n*=2,901)	1.03 (1.01 to 1.06)	0.013	1.03 (1.00 to 1.06)	0.030	1.00 (0.97 to 1.03)	0.773
Generalized FRS [2008] after excluding incident CVD (*n*=2,669)	1.03 (1.01 to 1.06)	0.017	1.03 (1.00 to 1.06)	0.049	0.99 (0.96 to 1.03)	0.651
Generalized FRS [2008] after excluding baseline depression (*n*=2,311)	1.05 (1.01 to 1.08)	0.005	1.05 (1.01 to 1.08)	0.010	1.01 (0.97 to 1.05)	0.668
Updated FRS [2015]^d^ (*n*=2,901)	1.10 (1.05 to 1.16)	<0.001	1.08 (1.02 to 1.14)	0.009	0.99 (0.93 to 1.07)	0.865

^a^Adjusted for sex

^b^Adjusted for sex, education, marital status, obesity, comorbidity burden, cognitive function

^c^Adjusted for sex, age, education, marital status, obesity, comorbidity burden, cognitive function

^d^Updated FRS algorithm https://www.thecalculator.co/health/Framingham-Risk-Score-Calculator-for-Coronary-Heart-Disease-745.html

### Sensitivity analyses

The results barely changed after excluding incident CVD cases (*n* = 323, Table 3). The results were also similar after excluding subjects with depression at baseline (*n* = 590). After adjusting for age, the associations again became non-significant. In Table 4, we present the association between FRS and the composite outcome of mortality and incident frailty. The findings remained the same.

**Table 4:** Associate between FRS at baseline (wave 1) and combined mortality and incident frailty during the follow-up (wave2-wave3)

	Model 1 ^a^		Model 2 ^b^		Model 3 ^c^	
	HR (95% CI)	*p* Value	HR (95% CI)	*p* Value	HR (95% CI)	*p* Value
Generalized FRS [2008] (*n*=3,033)	1.06 (1.04 to 1.09)	<0.001	1.05 (1.02 to 1.08)	<0.001	1.02 (0.99 to 1.05)	0.327
Generalized FRS [2008] after excluding incident CVD (*n*=2,794)	1.06 (1.03 to 1.09)	<0.001	1.05 (1.02 to 1.09)	<0.001	1.01 (0.98 to 1.05)	0.381
Generalized FRS [2008] after excluding baseline depression (*n*=2,407)	1.08 (1.04 to 1.11)	<0.001	1.07 (1.03 to 1.11)	<0.001	1.03 (1.00 to 1.07)	0.078
Updated FRS [2015]^d^ (*n*=3,033)	1.16 (1.11 to 1.21)	<0.001	1.13 (1.08 to 1.19)	<0.001	1.04 (0.98 to 1.10)	0.198

^a^Adjusted for sex

^b^Adjusted for sex, education, marital status, obesity, comorbidity burden, cognitive function score

^c^Adjusted for sex, age, education, marital status, obesity, comorbidity burden, cognitive function score

^d^Updated FRS algorithm https://www.thecalculator.co/health/Framingham-Risk-Score-Calculator-for-Coronary-Heart-Disease-745.html

## Discussion

Despite previous research showing an association between high risk of CVD and pre-frailty [29] or frailty [3], there are still knowledge gaps regarding the association between the FRS and frailty in older adults. The present study aimed to examine whether the FRS was useful in predicting future risk of frailty after accounting for chronological age. In this study, we found that the FRS was not independently associated with the development of frailty after adjusting for age. The FRS therefore failed to offer added value in predicting incident frailty beyond chronological age.

In this large population sample of Chinese older adults without a history of CVD at baseline, CVD risk factors including total cholesterol, systolic blood pressure, treatment for hypertension, smoking, and diabetes did not significantly differ by frailty status at baseline. In this study, high total cholesterol level was associated with lower risk of incident frailty (a.k.a. cholesterol paradox), which may be caused by survival bias [30]. Without adjusting for age, we found that higher FRS at baseline was associated with a higher risk of incident frailty during follow-up. Adjustment for potential confounding factors had little effect on the association, and the results remained essentially unchanged by excluding those who developed CVD during follow-up or had depression at baseline, which is consistent with the sex-specific results reported by others [17, 18]. However, the FRS and incident frailty relationship was lost when age was added to the multivariable model, suggesting that the age component of the FRS may be the primary driver of the relationship between FRS and incident frailty. This result is in agreement with one meta-analysis [31], which suggested that the FRS was no more strongly associated with future dementia than age. A cross-sectional analysis using data from the International Mobility in Aging Study found that frail older adults, compared to the non-frail, had higher FRS, and the association was independent of life course adversities (e.g., childhood social economic adversity) [32]. However, this cross-sectional study did not exclude prevalent CVD cases, which is a prerequisite for using the FRS to predict future health related outcome [4]. The non-significant association between diabetes and incident frailty in our cohort was a bit surprising. We found a strong positive association between CVD and diabetes at baseline. It is therefore possible that the reported association in the literature [33] between diabetes and frailty was partially due to confounding by CVD. In our study, diabetes was positively associated with incident frailty; the association however was not statistically significant. Therefore, the non-significance was likely due to the exclusion of prevalence cases of CVD at baseline.

To our knowledge, this is the first study to examine the longitudinal association between the FRS and incident frailty among older adults in China. Other strengths of our study include large sample size and the use of a well-validated frailty assessment. This study has several limitations. First, because the reported associations between FRS and incident frailty were limited to 4 years instead of 10 years, it is possible that a longer-term follow-up may reveal a positive relationship. Second, information on prevalent and incident CVD was based solely on self-report, this might have underestimated the actual CVD incidence, especially in rural areas [34, 35]. Third, not all participants at baseline provided a blood sample (a response rate of 67 %). Those who did not provide a blood sample tended to be man and urban residents [36], therefore introducing potential sample selection bias. Fourth, weight loss at baseline was defined as weight loss of 5 or more kilograms in the past year, instead of a loss of ≥ 10 % since last wave that was used at wave 2 and wave 3. However, the same criteria were used in previous studies [19, 22]. Fifth, compared to the analytic sample, those who were excluded due to missing data on FRS and frailty status were older, more likely to be non-smoker, and have less comorbidity burden and no diabetes (Table S1), which could introduce bias to the analysis. Finally, our findings might have been impacted by competing mortality, i.e., those who had higher Framingham score were more likely to die before frailty onset or whose frailty onset was not captured before death or dropout due to the discrete nature of the follow-up (i.e., every two years). But when we modeled incident frailty and mortality as a composite outcome, the association between FRS and combine outcome remained non-significant after adjusting for age.

## Conclusions

In summary, although CVD risk factors have been linked to frailty, this study found that the FRS was not associated with incident frailty independent of chronological age in Chinese older adults. It therefore offers no added value in predicting incident frailty. More studies are needed to understand the impact of CVD risk factors in the pathogenesis of frailty. In addition, further studies are necessary to clarify the predictive performance of FRS relative to chronological age in adverse health outcomes such as CKD [5], sarcopenia [6] and cognitive decline [7].

## Supplementary Information

**Additional file 1: Table S1.** Comparison of baseline characteristics of study subjects excluded due to missing data on FRS and frailty status and those sample included in the final analysis (*n*= 6,249)

## Data Availability

The raw data is available on website (http://charls.pku.edu.cn/).
