# Supplementary material for: The Framingham risk score is associated with incident frailty, or is it?
Source: BMC Geriatr. 2021 Jul 31;21:448. doi: 10.1186/s12877-021-02387-4 (PMC8325204; doi:10.1186/s12877-021-02387-4)
Supplement: Supplementary file 1 — Additional file 1: Table S1. Comparison of baseline characteristics of study subjects excluded due to missing data on FRS and frailty status and those sample included in the final analysis (n= 6,249) [file 12877_2021_2387_MOESM1_ESM.docx]

**Appendix**

Framingham Risk Score is Associated with Incident Frailty, or is It?

| Table S1. comparison of baseline characteristics of study subjects excluded due to missing data on FRS and frailty status and those sample included in the final analysis ( n= 6,249) | | | | |
| --- | --- | --- | --- | --- |
| Characteristic | Analytic sample |  | missing data on FRS and frailty | *P* Value |
|  | n=3,618(57.9) |  | n=2,631 (42.1) |  |
| Age (years), mean (SD) | 67.4 (6.5) |  | 69.2 (7.8) | <0.001 |
| Sex, n(%) |  |  |  |  |
| Male | 1,835 (50.7) |  | 1,352 (51.5) | 0.560 |
| Female | 1,783 (49.3) |  | 1,275 (48.5) |  |
| Total cholesterol (mg/dl), mean (SD) | 194.5 (38.5) |  | 192.6 (40.4) | 0.259 |
| HDL cholesterol (mg/dl), mean (SD) | 52.3 (15.7) |  | 50.9 (16.3) | 0.046 |
| SBP (mm Hg), mean (SD) | 135.1 (22.8) |  | 136.2 (23.5) | 0.147 |
| Antihypertensive treatment, n (%) |  |  |  |  |
| No | 2,870 (79.3) |  | 2,033 (79.4) | 0.933 |
| Yes | 748 (20.7) |  | 527 (20.6) |  |
| Smoking, n (%) |  |  |  |  |
| No | 2,467 (68.2) |  | 1,712 (71.9) | 0.002 |
| Yes | 1,151 (31.8) |  | 670 (28.1) |  |
| Diabetes, n (%) |  |  |  |  |
| No | 3,061 (84.6) |  | 2,340 (91.3) | <0.001 |
| Yes | 557 (15.4) |  | 222 (8.7) |  |
| Education, n (%) |  |  |  |  |
| Illiterate | 1,399 (38.7) |  | 959 (37.5) | <0.001 |
| Elementary school | 1,643 (45.4) |  | 1,072 (41.9) |  |
| Middle school | 410 (11.3) |  | 291 (11.4) |  |
| High school or above | 164 (4.5) |  | 237 (9.3) |  |
| Current residence, n (%) |  |  |  |  |
| Rural | 2,464 (68.1) |  | 1,418 (55.1) | <0.001 |
| Urban | 1,154 (31.9) |  | 1,155 (44.9) |  |
| Marital status, n (%) |  |  |  |  |
| Married | 2,899 (80.1) |  | 1,944 (75.6) | <0.001 |
| Divorced | 47 (1.3) |  | 35 (1.4) |  |
| Widowed | 638 (17.6) |  | 562 (21.7) |  |
| Never married | 34 (0.9) |  | 30 (1.2) |  |
| Obese, n (%) |  |  |  |  |
| No | 3,301 (92.7) |  | 1,285 (93.2) | 0.575 |
| Yes | 259 (7.3) |  | 94 (6.8) |  |
| Cognitive function^b^, mean (SD) | 10.2 (4.2) |  | 10.3 (4.4) | 0.157 |
| Depressive symptoms scores ^c^, mean (SD) | 7.7 (5.3) |  | 7.4 (5.4) | 0.048 |
| Comorbidity burden ^d^, mean (SD) | 1.3 (1.3) |  | 1.0 (1.2) | <0.001 |
| Abbreviations: *SBP* systolic blood pressure, *CVD* cardiovascular disease, *SD* standard deviation, *HDL* high density lipoprotein cholesterol | | | | |
| ^b^ Cognitive function was measured by the modified mini-mental status examination | | | | |
| ^c^ Depressive symptoms was measured by the 10-item Center for Epidemiologic Studies Depression Scale | | | | |
| ^d^ Comorbidity includes cancer[excluding minor skin cancers], chronic lung diseases, liver disease, kidney disease, stomach or other digestive disease, arthritis/rheumatism and asthma. | | | | |
